# Investigating the effects of make-up water dilution and oil presence on polymer retention in carbonate reservoirs

**DOI:** 10.1038/s41598-024-78743-2

**Published:** 2024-11-13

**Authors:** Anoo Sebastian, Muhammad Mushtaq, Emad Walid Al-Shalabi, Waleed AlAmeri, Kishore Mohanty, Shehadeh Masalmeh, Ali M. AlSumaiti

**Affiliations:** 1 Chemical and Petroleum Engineering Department, SAN Campus, Khalifa University of Science and Technology, Abu Dhabi, UAE; 2 Research and Innovation Center on CO2 and Hydrogen (RICH), Department of Chemical and Petroleum Engineering, Khalifa University of Science and Technology (KU), Abu Dhabi, UAE; 3 Hildebrand Department of Petroleum and Geosystems Engineering, The University of Texas at Austin, Austin, USA; 4 Abu Dhabi National Oil Company (ADNOC), Abu Dhabi, UAE

**Keywords:** Acrylamido-Tertiary-Butyl Sulfonate (ATBS)-based polymers, Polymer retention, Low salinity polymer flooding, Carbonate reservoirs, Chemical engineering, Crude oil

## Abstract

The application of polymer flooding is challenging in harsh temperature and salinity conditions in Middle-Eastern carbonate reservoirs, as they can deteriorate the commonly used polymers such as Hydrolyzed Polyacrylamide (HPAM). One solution to this issue is the use of newly developed Acrylamido-Tertiary-Butyl Sulfonate (ATBS) based polymers, which can endure adverse temperature and salinity conditions. However, they also tend to adsorb onto carbonate rocks with positive surface charge. This study aims to tackle the problem of high polymer retention by employing low-salinity polymer flooding. For that coreflooding experiments were conducted on an ATBS-based polymer in salinities ranging from 400 to 167,000 ppm using fully water-saturated cores and cores at residual oil saturation (S_or_). The single-phase retention experiments determined polymer retention values of around 25 µg/g-rock when using diluted brines, which is about half of the retention values (47–56 µg/g-rock) observed with high salinity seawater (43,000 ppm) and formation water (167,000 ppm). Furthermore, the retention of the ATBS-based polymer was further reduced by 50% in the presence of oil compared to the experiments conducted in the absence of oil. The results demonstrated that an optimal salinity threshold of 10,000 ppm and lower yields significant improvements in the efficiency of polymer flooding.

## Introduction

The global energy demand has increased significantly over the past few decades, driven by population growth, urbanization, and industrialization. Despite the boom of renewable energy sources, hydrocarbons continue to play a decisive role in meeting this demand. Therefore, enhanced oil recovery (EOR) techniques have become vital to maximize production from existing reservoirs. Moreover, EOR techniques support sustainable oil production by facilitating more efficient recovery from existing reservoirs. Polymer flooding, a chemical EOR method, is a proven technique that has been successfully implemented during the initial stages of waterflooding^1–3^. The process involves introducing polymers to enhance injection water viscosity, thereby improving the water-oil mobility ratio, reducing viscous fingering, and enhancing water sweep efficiency^4–6^. By improving sweep efficiency, the amount of water produced along with the oil is reduced, leading to cost savings and lower environmental impact. Additionally, polymer flooding reduces the time needed to produce the same amount of oil compared to waterflooding. Faster oil production means the entire process becomes more energy-efficient. The equipment runs for a shorter time, leading to less energy consumption and lower CO_2_emissions^7,8^.

While polymer flooding is an effective technique for enhancing oil recovery, it encounters several challenges in carbonates in the Middle East due to the existing harsh conditions of high temperature and high salinity. The complex conditions of these carbonate reservoirs include heterogeneity, mixed-to-oil wettability, low permeability (< 100 mD), high salinity (> 100,000 ppm) and hardness (> 10,000 ppm), and high temperature (> 85^o^C)^9–11^. As a result, the use of conventional polymers such as synthetic Hydrolyzed Polyacrylamide (HPAM) and Xanthan gum for polymer flooding in carbonate reservoirs is limited. The HPAM polymers pose stability issues at high temperatures and salinity environments and have a higher affinity for positively charged carbonate rock surfaces^10,12^. In contrast, Xanthan gum, a biopolymer, is less affected by salinity. Nevertheless, it exhibits low injectivity when employed in carbonate rocks, especially with low permeability^13^.

In response to the challenges posed by high salinity and temperature in carbonate reservoirs, new acrylamide-based polymers have been developed. These polymers integrate unique monomers, such as N-vinylpyrrolidone (NVP) and Acrylamido-Tertiary-Butyl Sulfonate (ATBS), to improve their chemical stability^14^. NVP is a non-ionic water-soluble monomer with a low retention rate, rendering it ideal for carbonate reservoirs. ATBS-based polymers are anionic polymers that are effective in enhancing oil recovery at high temperatures and salinity conditions^12^. However, these novel polymers are more expensive than other commercially available options, and their retention may affect the performance of the polymer flooding process^5,15^.

Polymers are retained in several ways, including adsorption, mechanical entrapment, and hydrodynamic retention. The most prevalent mechanism is adsorption, where synthetic polymers are attracted to reservoir rocks through electrostatic forces, and van der Waals forces, while biopolymers are attracted through hydrogen bonding forces^16,17^. Mechanical entrapment is observed in low-permeability reservoirs, where polymer molecules become trapped in pore spaces with small throat sizes^18–20^. The process of hydrodynamic retention is reversible and has minimal impact. It is dependent on the flow rate of the polymer solution^21–23^.

The level of polymer retention can significantly affect the project’s economics. If the retention levels are too high, more polymer may be required to achieve the desired viscosity, leading to a considerable increase in project costs^5,15^. Moreover, the trapped polymer can reduce the efficacy of the polymer flooding process by blocking the pores of the rock and reducing the mobility of the injected fluid^13^.

The injection of low-salinity water is a promising method to decrease polymer retention in polymer flooding projects^24,25^. The exact mechanism by which low-salinity polymer flooding leads to decreased retention remains unclear. In the literature, it has been reported that when exposed to low-salinity brine, the hydrodynamic size of the polymer increases, and fewer molecules are needed to occupy the adsorption sites. This ultimately leads to a reduction in polymer retention^26^. On the other hand, several studies have reported that low-salinity polymer flooding causes injectivity issues due to the dynamic behavior of the polymer in low-salinity brine^27^. The hydrodynamic size of the polymer in low-salinity brine can affect the polymer flooding by clogging the rock’s pores, reducing the reservoir’s permeability, and increasing the injection pressure^27^.

Nevertheless, low-salinity polymer injection offers several advantages over high-salinity schemes. The overall economics of polymer flooding is influenced by several factors, including the amount of oil recovered, polymer cost, polymer injection costs, polymer production costs, and the treatment costs for both water and produced fluids. The major cost involved is the price of polymer, and for commonly used HPAM polymers, the cost is $1.50/lb^28,29^. Typically, polymer flooding involves operational costs of approximately $0.58 per barrel higher than waterflooding^30^.

Low-salinity polymer flooding can reduce polymer flooding project costs in multiple ways. Lower salinity brine decreases the required polymer concentration to achieve the desired viscosity, especially in high-salinity reservoirs. For instance, in reservoirs with 260,000 ppm TDS, polymer concentration can be reduced by 3–4 times by lowering brine salinity to 1,500 ppm TDS. Additionally, reduced shear sensitivity at lower salinity helps minimize viscosity loss during mechanical processes. Lower salinity also reduces issues such as scaling, souring, and oil/water separation, improving operational efficiency and cutting related costs. Minimizing polymer adsorption reduces the delay in oil recovery and further lowers overall expenses. These cost savings are even more significant in harsh reservoirs where more expensive polymers are required^29^.

However, in low-salinity polymer flooding, the production of low-salinity brine involves certain costs. Reverse osmosis (RO) desalination costs are approximately $0.13/bbl for treated water onshore and over $0.35/bbl offshore^31^. Onshore treatment costs for produced water in the North Sea range from $0.19 to $3.40 per barrel^32^.

Ayirala et al. conducted an advanced facility engineering analysis that assessed the cost-effectiveness of using low-salinity water for offshore polymer flooding compared to traditional seawater polymer flooding. The study explored two scenarios with polymer solution viscosities of 3 cP and 6 cP. The findings revealed that low-salinity water polymer flooding is more cost-effective than seawater polymer flooding. The additional investment in desalination technology could be recovered within 1.6 to 4 years, primarily due to significant cost savings in chemical consumption and polymer processing. Furthermore, the payback period was shorter for higher-viscosity polymer solutions, which typically require greater polymer concentrations and larger facilities when using seawater, resulting in considerable operational cost savings^33^.

This study aims to enhance the understanding of polymer performance in low-salinity brine within porous media. It focuses on diluting representative formation water and seawater under Middle Eastern carbonate reservoir conditions to analyze the performance of an ATBS-based polymer. There is a lack of research on the effectiveness of low-salinity polymer flooding, particularly for carbonate rocks using advanced ATBS polymers. This study seeks to identify the minimum salinity level at which promising outcomes, such as decreased polymer usage and retention, can be attained. The results of this research could contribute to the effective implementation of low-salinity polymer flooding in carbonate reservoirs.

## Materials

The materials used for this study included a range of different synthetic brines, which consisted of formation water (FW) and its dilutions, namely FW-1 (formation water diluted 5 times), FW-2 (formation water diluted 10 times), FW-3 (formation water diluted 20 times), and seawater (SW) and its dilutions, namely SW-1 (seawater diluted 25 times) and SW-2 (seawater diluted 100 times). In the case of formation water (167,114), the dilution levels were selected to have a range of salinities that will be used in our future oil recovery studies involving low-salinity brines. The dilutions were chosen for seawater (42,507) based on their potential to enhance oil recovery by low-salinity effect in the carbonate reservoir^34^. Both formation water and seawater are representative of the Middle Eastern reservoir conditions, and dilutions of both formation water and seawater are being considered so that the study can be applied to both onshore and offshore reservoirs, respectively. Also, when comparing the ionic compositions of formation water and seawater (Tables 1 and 2), seawater contains sulfate ions, while formation water lacks any sulfate content. This is expected to play a role in investigating dilutions effects on oil recovery from carbonates in future studies. Additionally, a representative sample of crude oil from the Middle East was used for the study. Moreover, SAV10, an ATBS-based polymer of molecular weight 4.3–6.8 MDa, provided by SNF Floerger, was used as a key component in the study.

Indiana limestone outcrop samples of 3-inch length and 1.5-inch diameter with similar permeabilities were utilized for the coreflooding experiments. Carbonate rocks are sedimentary rocks primarily composed of minerals such as calcite (CaCO_3_), dolomite (CaMg(CO_3_)_2_), and anhydrite (CaSO_4_). However, most carbonate oil and gas reservoirs are predominantly rich in calcite^35^. Indiana limestone outcrops contain 97.7% calcite mineral^36^, hence can be a reasonable representative of carbonate cores. These materials were carefully selected to ensure accuracy and reliability in the results obtained from the study. Tables 1 and 2 present the ionic compositions of the seven synthetic brines used, while Table 3 provides the fluid properties at ambient conditions (25^o^C). The petrophysical characteristics of the core samples and the experimental parameters applied are detailed in Table 4. The experimental temperature of 25^o^C was selected based on one of our previous studies, which demonstrated that the retention of the specific polymer used in this research was not significantly affected by temperature^37^.

**Table 1:** Ionic composition of formation water and its different dilutions.

Ionic Composition	FW (ppm)	FW-1 (ppm)	FW-2 (ppm)	FW-3 (ppm)
Na^+^	52,952	10,590	5,295	2,648
Ca^2+^	9,250	1,850	925	463
Mg^2+^	1,446	289	145	72
K^+^	744	149	74	37
Cl^−^	102,722	20,544	10,272	5,136
**TDS**	**167**,**114**	**33**,**423**	**16**,**711**	**8**,**356**

**Table 2:** Ionic composition of seawater and its different dilutions.

Ionic Composition	SW (ppm)	SW-1 (ppm)	SW-2 (ppm)
Na^+^	13,072	523	131
Ca^2+^	539	22	5
Mg^2+^	1,583	63	16
K^+^	498	20	5
Cl^−^	23,517	941	235
SO_4_^2−^	3,298	132	33
**TDS**	**42**,**507**	**1**,**701**	**425**

## Methodology

**Bulk-Rheology Experiments**. An Anton Paar MCR 302 was used to conduct the rheological experiments on polymer stock solutions and their dilutions in the formation water and seawater at 25^o^C. The polymer concentration required in formation water and seawater to achieve the desired viscosity (4.5 cP) was determined by plotting the polymer concentration versus viscosity at a shear rate of 10 s^−1^.

**Table 3:** Fluid properties at 25^o^C.

Fluid	Density (g/cc)	Viscosity (cP)
FW	1.11	1.20
FW-1	1.02	1.01
FW-2	1.01	1.00
FW-3	1.00	0.90
SW	1.03	1.02
SW-1	1.00	0.89
SW-2	1.00	0.89
Crude Oil	0.84	5.00

**Table 4:** Petrophysical characteristics of the cores and experimental parameters applied for coreflooding experiments.

Core ID (Single-phase Tests)	CF-1	CF-2	CF-3	CF-4	CF-5	CF-6	CF-7
Porosity (%)	17	15	15	15	16	16	17
Brine Permeability (mD)	265	265	286	283	244	231	225
**Core ID (Two-phase Tests)**	**CF-8**	**CF-9**	**CF-10**	**CF-11**	**CF-12**	**CF-13**	**CF-14**
Porosity (%)	15	15	15	15	15	19	15
Brine Permeability (mD)	200	224	284	263	218	260	223
Initial Water Saturation (S_wi_) (%)	32	33	32	34	36	32	34
Residual Oil Saturation (S_or_) (%)	35	34	35	35	38	31	39
Connate Water/ Injection Water Salinity, TDS (ppm)	167,114 FW	33,423 FW-1	16,711 FW-2	8,356 FW-3	42,507 SW	1,700 SW-1	425 SW-2
Polymer Concentration (ppm)	1000						
Temperature (^o^C)	25						
Confining Pressure (psi)	1200						
Back Pressure (psi)	100						

**Dynamic Adsorption Experiments**. The experiments were conducted using core samples completely saturated with brine and core samples at residual oil saturation (S_or_). The objective was to study the adsorption of the polymer during polymer injection, both in the presence and absence of oil. The experiments were performed at 25^o^C, confining pressure of 1200 psi and backpressure of 100 psi (Table 4). To conduct polymer adsorption studies, the samples underwent a brine pre-flush at a constant flow rate of 0.5 cc/min until the pressure was stable. Following brine injection, a slug of the polymer solution was introduced at a constant flow rate of 0.5 cc/min. Afterward, an extended post-flush brine injection at the same flow rate of 0.5 cc/min was carried out to remove all mobile and unadsorbed polymer from the core. It is worth noting that the experiments were conducted using a 0.5 cc/min rate to reduce the time needed for adsorption equilibrium. The effluent polymer solution was collected at regular intervals to determine the polymer retention level. Polymer concentration was measured using the UV method in single-phase experiments and the TOC-TN method in two-phase experiments. Further, normalized polymer concentration (C_e_/C_i_) was plotted against injected pore volumes (PV), and the material balance equation, i.e., Eq. (1), was applied to calculate the polymer retention.

1 $$\:{A}_{d}=\frac{({C}_{i}{V}_{i}-{\sum\:}_{i=1}^{n}{C}_{e}{V}_{e})}{{W}_{d}}$$

where A_d_ is the polymer adsorption (µg/g of rock), C_i_ and V_i_ are the initial concentration (mg/l) and volume (ml), respectively, C_e_ and V_e_ are the effluent concentration (mg/l) and volume (ml) in each vial, respectively, and W_d_ is the dry weight of the core sample (g).

*Residual Resistance Factor.* The residual resistance factor (RRF) measures the resistance to flow in porous media after polymer flooding. The RRF was calculated using Eq. (2), respectively:

2 $$\:\text{R}\text{R}\text{F}=\frac{{\varDelta\:\text{P}}_{brine\:post-flush}}{{\varDelta\:\text{P}}_{brine\:pre-flush}}$$

where ΔP_brine pre−flush_, ΔP_brine post−flush_ are the pressure drop data (psi) recorded during brine pre-flush and brine post-flush, respectively.

*Polymer Adsorbed Layer Thickness.*Polymer adsorbed layer thickness refers to the thickness of the layer of polymer molecules that adheres to the rock surface (pore walls) during the polymer flooding process. It was determined by calculating the reduction in permeability under the assumption of Poiseuille fluid flow through a capillary constricted by a uniform layer of polymer^38^, as depicted in Eqs. (3) and (4).

3 $$\:e={r}_{p}\:(1-\frac{1}{RR{F}^{\frac{1}{4}}})$$

4 $$\:{r}_{p}={\left(\frac{8*K}{\phi}\right)}^{1/2}$$

where $\:e$ is the adsorbed polymer layer thickness (µm), r_p_ is the average pore radius for water flow (µm), *K*is the brine permeability (µm^2^), and $\phi$ is the porosity (fraction).

## Results and discussion

**Bulk-Rheological Studies.** This section describes the results obtained for polymer bulk viscosities using different water dilutions, shear rates, and polymer concentrations.

**Fig. 1:**
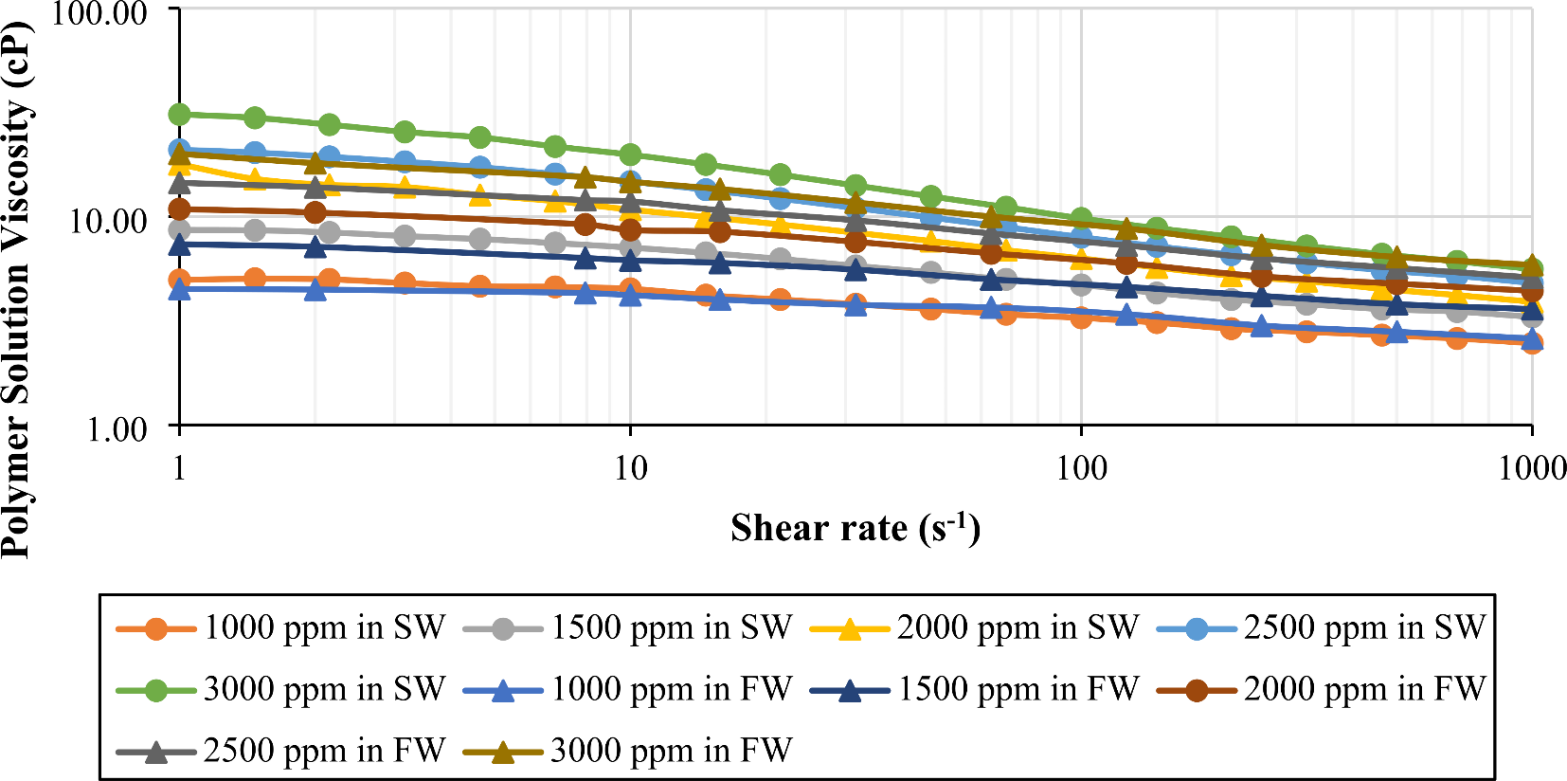
Bulk-rheology of polymer in SW (42, 507 ppm) and FW (167,114 ppm) at 25^o^C. The figure was created using Microsoft Excel (Product name: Microsoft 365, Version number: Version 2409 (Build 18025.20104, 64-bit)).

*Shear Ramp-up Studies.* The data presented in Fig. 1depicts the behavior of polymer solutions in the rheometer concerning their bulk viscosity and shear rate. The viscosity was measured by varying polymer concentrations (1000 to 3000 ppm) in both formation water and seawater. One can observe that all polymer solutions exhibited shear-thinning behavior; the viscosity decreases as the shear rate increases. This behavior is mainly caused by the deformation of the polymer molecules under high shear rates. When the shear rate increases, the energy input causes the polymer molecules to deform and align in the flow direction (rotatory). This process reduces the hydrodynamic radius of the polymer chains and subsequently decreases the solution’s viscosity by lowering the resistance to flow. The degree of shear-thinning is more noticeable in solutions with higher polymer concentrations (3000, 2500, and 2000 ppm) compared to those with lower polymer concentrations (1500 and 1000 ppm). This is because high polymer concentrations tend to result in stronger entanglements and higher viscosity. Durinin the shearing process, the induced alignments of molecules show significantly decreased viscosity, leading to a more pronounced shear-thinning behavior^39,40^.

**Fig. 2:**
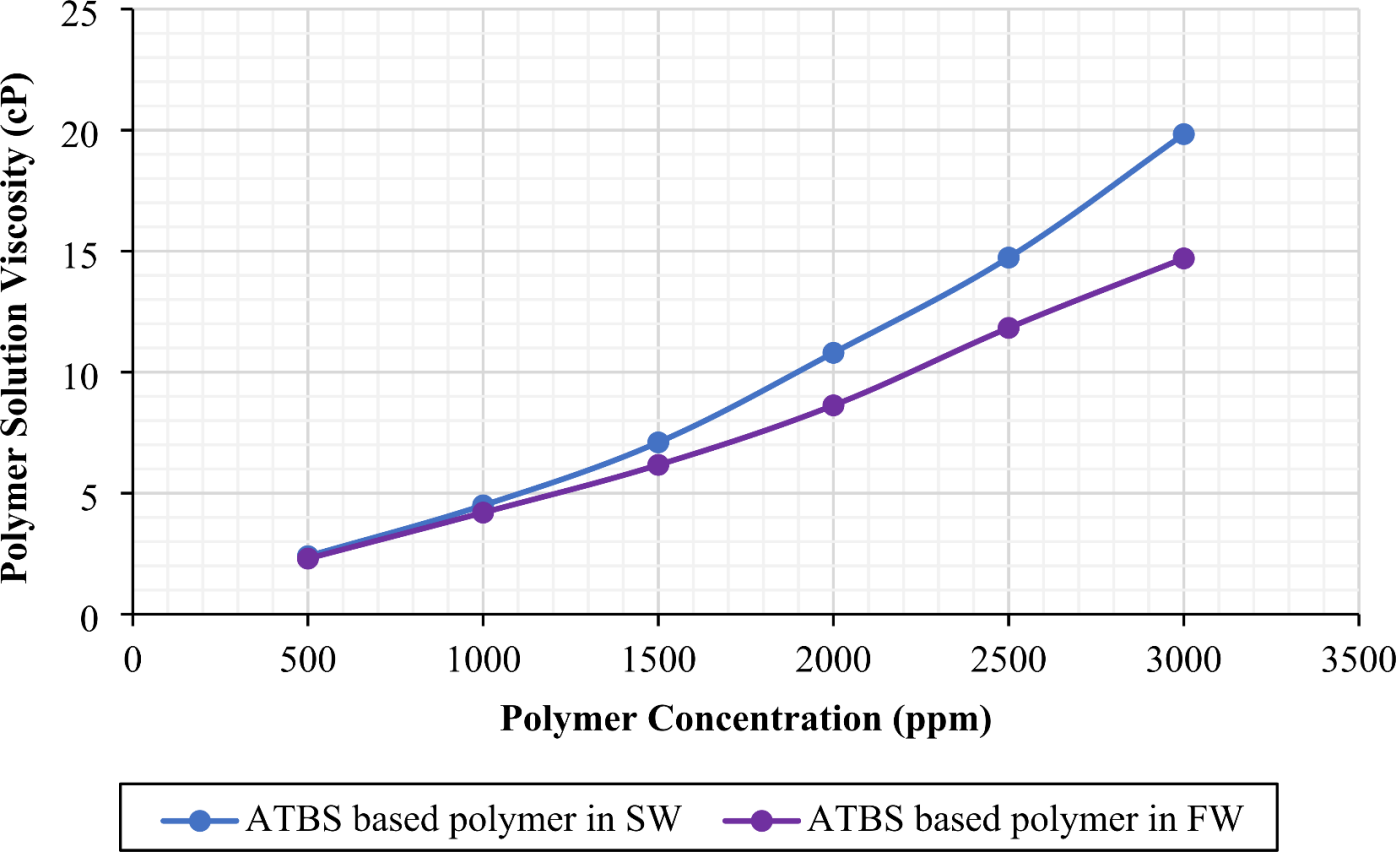
Polymer concentration vs. polymer solution viscosity. The figure was created using Microsoft Excel (Product name: Microsoft 365, Version number: Version 2409 (Build 18025.20104, 64-bit)).

*Effect of Polymer Concentration on Polymer Solution Viscosity.* The effect of polymer concentration on solution viscosity in formation water and seawater is shown in Fig. 2. The viscosity of the solution increases as the polymer concentration increases. The reason for an increase in solution viscosity is due to the interaction between polymer chains through intermolecular forces. When the concentration of polymer increases, there are more polymer chains in the solution, which results in increased intermolecular interactions and greater entanglement density of the polymer chains. As a result, the solution’s viscosity rises due to higher resistance to flow^41–43^. Based on the data in Fig. 2, the ideal polymer concentration needed to achieve a viscosity of 4.5 cP at a shear rate of 10 s^−1^ and a temperature of 25^o^C is 1000 ppm for polymer solution in both formation water and seawater. A shear rate of 10 s^−1^was chosen as it corresponds to a reservoir flow rate of 1 ft/day^44^. Additionally, a target viscosity of 4.5 cP was considered to achieve the minimum total relative mobility of oil and water phases during the seawater (43,000 ppm) injection cycle under reservoir conditions^25,45^. Also, this polymer viscosity is needed to address the permeability contrast between two zones in representative Middle Eastern carbonate reservoirs^46,47^. Further, a constant concentration of 1000 ppm was used for all the brine formulations to evaluate the effects of water salinity on the performance of the ATBS-based polymer. The concentration was kept constant to investigate the effect of salinity on polymer solution viscosity and adsorption, as polymer concentration is a key factor influencing both viscosity and adsorption behaviors^48^.

**Fig. 3:**
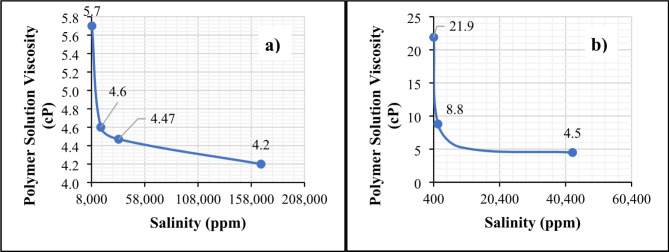
Polymer solution viscosity vs. make-up water salinity: (a) Formation water and its dilutions, (b) Seawater and its dilutions. The figure was created using Microsoft Excel (Product name: Microsoft 365, Version number: Version 2409 (Build 18025.20104, 64-bit)).

*Effect of Make-up Water Dilution on Polymer Solution Viscosity*. Figure 3 shows the effect of make-up water salinity on the bulk viscosity of polymer solutions in different brines. The findings indicate that polymer viscosity did not increase significantly in brine solutions with salinities of 33,423 and 16,711 ppm (5 times and 10 times diluted formation water) (Fig. 3a). The viscosity of polymer solutions increased significantly when mixed with water that had lower salinity levels (below 10,000 ppm), which includes brine solutions with 20 times diluted formation water (FW-3) and 25-times (SW-1), and 100-times diluted seawater (SW-2) with salinity levels of 8,356 ppm, 1,701 ppm, and 425 ppm, respectively. The viscosity factors increased by a range of 1.2 to 5.0, as shown in Fig. 3a and b^49^. This increase in polymer solution viscosity is due to the pronounced repulsive intermolecular forces between the anionic backbone chain of the polymer molecules at low salinity levels (less ionic species) that expand the molecular chains, straighten them up, and swell the solution^50,51^, leading to a larger hydrodynamic size^52^. Conversely, the increased ionic strength at higher salinities reduces this electrostatic repulsion, causing the polymer chains to coil and aggregate, resulting in a smaller hydrodynamic size^53–55^ and lower viscosity. Equation (5)^56,57^ represents an analytical expression relating the hydrodynamic radius and viscosity of polymer solution.

5 $$\:{R}_{h}={\left(\frac{3}{10\pi\:{N}_{A}}\right)}^{1/3}({\mu\:{M}_{w})}^{1/3}$$

where R_h_ is the hydrodynamic radius of a rigid sphere of a flexible polymer in solution (m), N_A_ is the Avogadro’s number (mol^−1^), µ is the intrinsic viscosity of the polymer solution (cm^3^/g), and M_w_ is the molecular weight of the polymer (MDa).

A similar study conducted by Vermolen et al. examined various polymers, specifically partially hydrolyzed polyacrylamide (HPAM) based co- and ter-polymers functionalized with 2-Acrylamido-2-Methylpropane Sulfonate (AMPS) and n-Vinyl Pyrrolidone (NVP) monomers, prepared in brines with different salinity levels (241 ppm, 43,730 ppm, 179,853 ppm, and 200,000 ppm). The findings revealed that the viscosifying power of these polymers decreases as brine salinity increases and is attributed to the increased screening of the polymer charges at higher salt concentrations^58^.

**Fig. 4:**
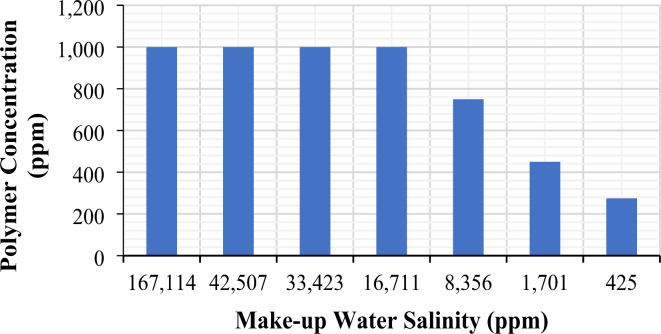
Polymer concentration for the seven brines (FW and SW dilutions) needed to achieve a targeted viscosity of 4.5 cP. The figure was created using Microsoft Excel (Product name: Microsoft 365, Version number: Version 2409 (Build 18025.20104, 64-bit)).

Figure 4, taken from our previous study^49^, displays the polymer concentrations necessary to attain the desired viscosity of 4.5 cP at a temperature of 25^o^C and a shear rate of 10 s^[-1^for different brines (FW and SW dilutions). The findings show that when the salinity level is at 8,356, 1,701, and 425 ppm (which corresponds to 20-times diluted formation water, 25-times diluted seawater, and 100-times diluted seawater, respectively), the amount of polymer required to achieve the desired viscosity decreases significantly by 25%, 55%, and 73%, respectively. When polymer solutions are prepared in low salinity, they show an increase in viscosity, implying that a lower amount of polymer is needed to achieve the desired viscosity. As explained above, the anionic nature of the polymer and the prevalence of repulsive forces at lower salinities cause the polymer molecular chains to expand, increasing the hydrodynamic size of the polymer. Consequently, a lower polymer concentration can achieve the required viscosity, reducing the amount of polymer needed and the associated costs^59,60^. However, in the current study, the polymer concentration was maintained at a constant 1000 ppm across all salinities to examine the effect of salinity on both polymer viscosity and retention behaviors.

**Dynamic Polymer Retention Studies**. In order to ensure precise polymer retention measurements, 14 dynamic retention experiments were conducted, 7 without oil (single-phase) and 7 with oil present (two-phase). In the single-phase experiments, the adsorption tests were conducted by first injecting brine, then polymer, and finally flushing with brine at a rate of 0.5 cc/min. In the brine post flush stage, approximately 60–70 pore volumes of brine were injected to ensure complete flushing out of the polymer. During the polymer injection and brine post-flush, effluents were collected every 0.2 PV for the first 5 PVs. Once the differential pressure stabilized, the collection interval was increased to 1 PV.

**Fig. 5:**
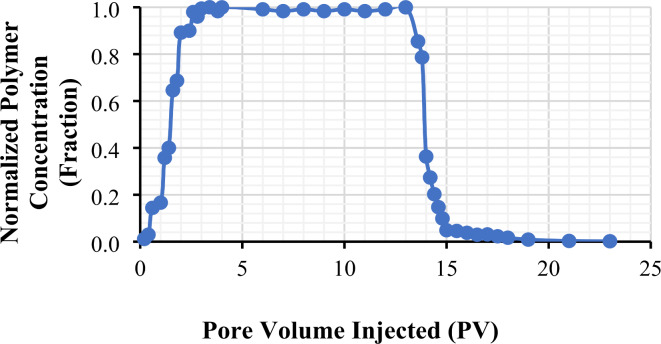
Polymer concentration profile of CF-1 during polymer injection and brine post-flush cycles at 25 °C. The figure was created using Microsoft Excel (Product name: Microsoft 365, Version number: Version 2409 (Build 18025.20104, 64-bit)).

On the other hand, for the two-phase experiments, the oil was injected first to bring the core to S_wi_ condition, then aged at 90^o^C for 14 days to alter wettability to non-water wet. Next, glycerin brine solution was injected to force imbibition and bring the cores to S_or_ condition, thereby removing the maximum amount of mobile oil from the sample. This helped prevent any oil mobilization during polymer injection and allowed for better characterization of polymer retention. Polymer retention studies at S_or_ condition were performed after flushing the glycerin out of the core with brine. The polymer concentration was analyzed in collected effluent samples at regular intervals, and the results were plotted as shown in Fig**. **5^49^ as a typical normalized profile.

*Effect of Make-up Water Dilution on Dynamic Polymer Retention in the Absence and Presence of Oil.*In the single-phase experiments, the material balance method yielded dynamic polymer retention values of 56 and 47 µg/g-rock for the base cases of formation water and seawater, respectively^49^. The amount of polymer retention varied when different formation water dilutions were used. The dynamic polymer retention values were 50 µg/g-rock for 5-times diluted formation water with 33k ppm salinity, 46 µg/g-rock for 10-times diluted formation water with 16k ppm salinity, and 25 µg/g-rock for 20-times diluted formation water with 8k ppm salinity. The first two dilutions of FW-1 and FW-2 did not show a pronounced difference in polymer retention, but the third dilution of FW-3 showed a significant reduction. This suggests that reducing salinity below 10k ppm is necessary to achieve a notable reduction in polymer retention. In addition, a significant reduction in polymer retention was observed when SW was diluted 25 times (1,701 ppm) and 100 times (425 ppm) with values of 38 and 24 µg/g-rock, respectively. It is interesting to highlight that both salinities were below 10,000 ppm, which confirms the previous observation that there were notable reductions in polymer retention at salinities below 10,000 ppm. The reduction in polymer retention with decreasing water salinity is justified by the high solvency of low salinity water for the polymer, which reduces the interactions between the polymer and the rock. Additionally, at reduced salinities, the repulsive forces between the negatively charged polymer chains cause them to expand and adopt a more extended conformation in the solution, resulting in a larger hydrodynamic size of the polymer molecules and increased solution viscosity. As a result, fewer polymer molecules are required to occupy the available adsorption sites, leading to lower overall adsorption. Furthermore, the polymer chains expand in low-salinity brine, resulting in unfavorable conformations for adsorption and high conformational entropy loss during adsorption^26,27^.

For the two-phase experiments, significantly lower polymer retention values were observed for CF-8 to CF-14 compared to those in the single-phase experiments (CF-1 to CF-7). The retention values for CF-8 to CF-14 were 26, 26, 23, 19, 28, 20, and 14 µg/g-rock, respectively as opposed to the values in the single-phase experiments (CF-1 to CF-7) of 56, 50, 46, 25, 47, 38, and 24 µg/g-rock, for the respective brines of FW, FW-1, FW-2, FW-3, SW, SW-1, and SW-2. These low polymer adsorption values are due to the presence of oil and the relatively more oil-wet cores, which significantly reduced available rock surface area for polymer molecules adsorption.

**Fig. 6:**
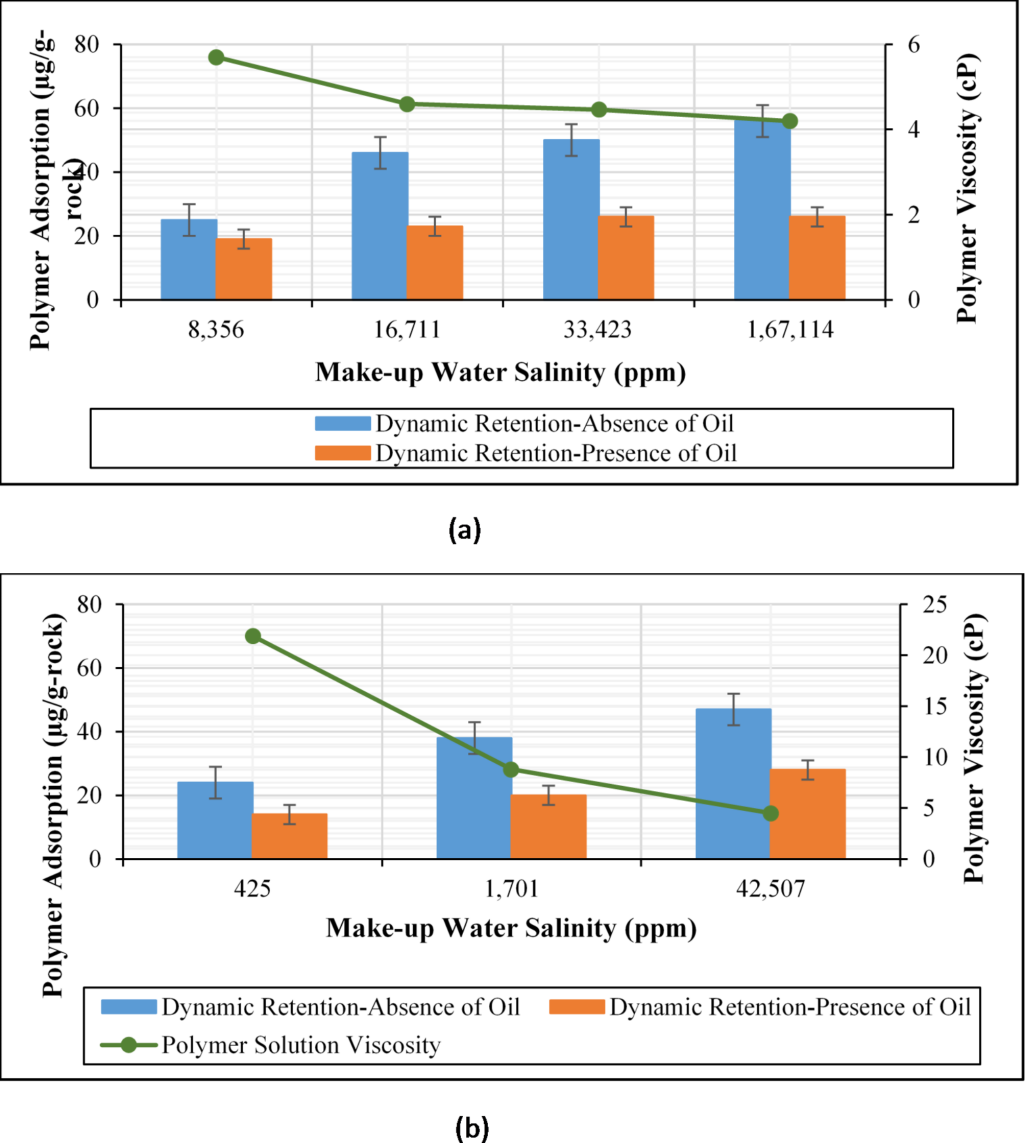
(a) Dynamic polymer retention for formation water and its dilutions. The figure was created using Microsoft Excel (Product name: Microsoft 365, Version number: Version 2409 (Build 18025.20104, 64-bit)). (b) Dynamic polymer retention for seawater and its dilutions. The figure was created using Microsoft Excel (Product name: Microsoft 365, Version number: Version 2409 (Build 18025.20104, 64-bit)).

The impact of salinity on the polymer adsorption onto the cores in the presence of oil was also noted. When low salinity brines (8k, 1,701, and 425 ppm) were used, polymer retention values were lower (19, 20, and 14 µg/g-rock) compared to the values observed with formation water (167k ppm) and seawater (43k ppm), which resulted in higher retention values of 26 and 28 µg/g-rock, respectively. The low salinity of the brine used in the two-phase experiments contributed to the observed lower retention values. Additionally, as stated above, the core’s preferably oil-wet nature and the presence of oil led to a reduction in the rock surface area available for polymer adsorption^61–64^. Figure 6a and b presents the dynamic polymer retention results for all coreflooding experiments. One should note that polymer concentration was measured using different methods for single- and two-phase experiments. UV-vis spectroscopy was used for the single-phase experiments, while the TOC-TN method was used for the two-phase experiments. By applying the theory of error propagation, uncertainties of ± 5 µg/g-rock and ± 3 µg/g-rock were observed for the single-phase and two-phase experiments, respectively^37^.

**Fig. 7:**
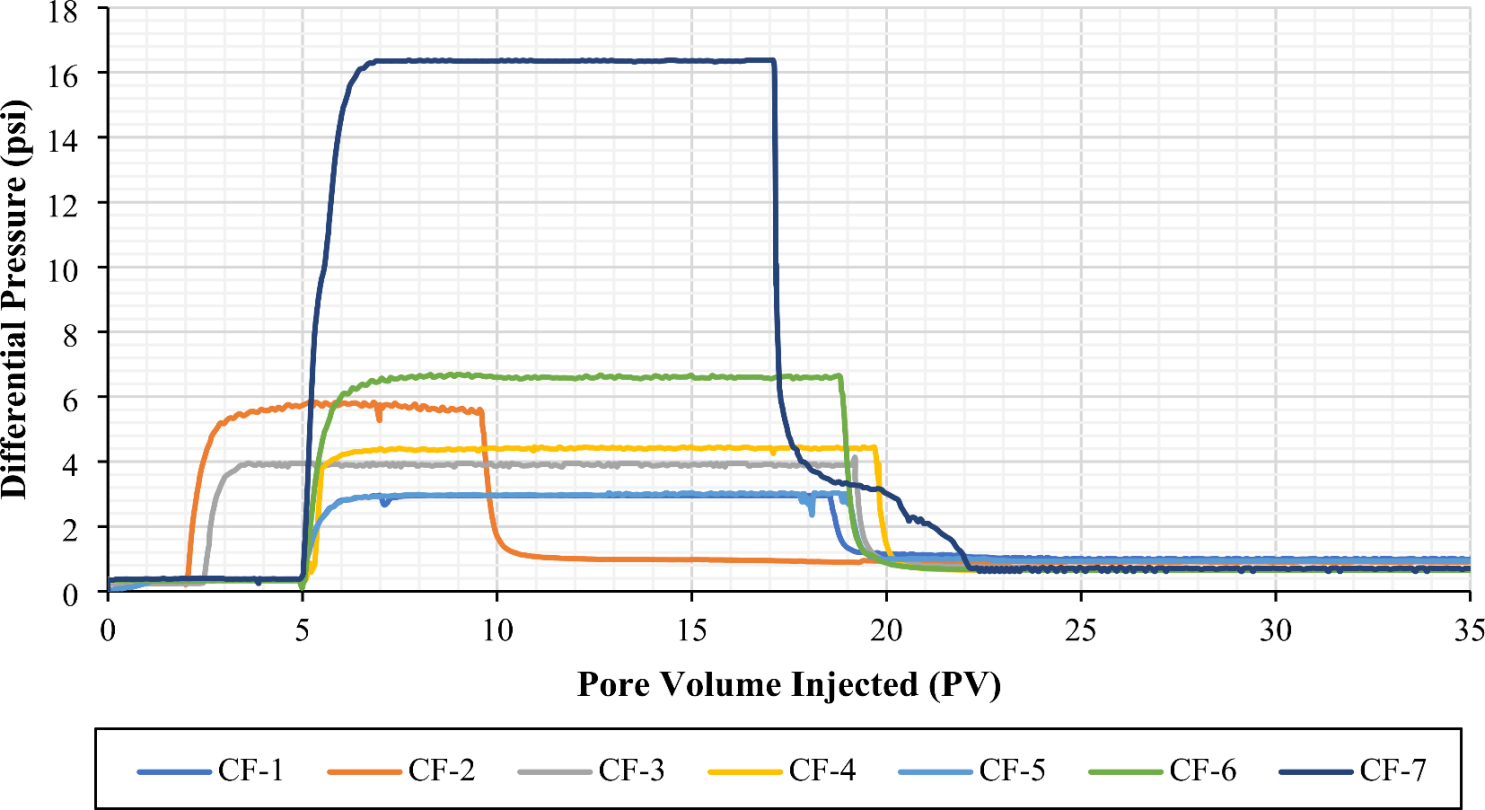
Differential pressure profile for brine pre-flush, polymer injection, and brine post-flush for CF-1 to CF-7 (Single-phase coreflooding experiments). The figure was created using Microsoft Excel (Product name: Microsoft 365, Version number: Version 2409 (Build 18025.20104, 64-bit)).

**Fig. 8:**
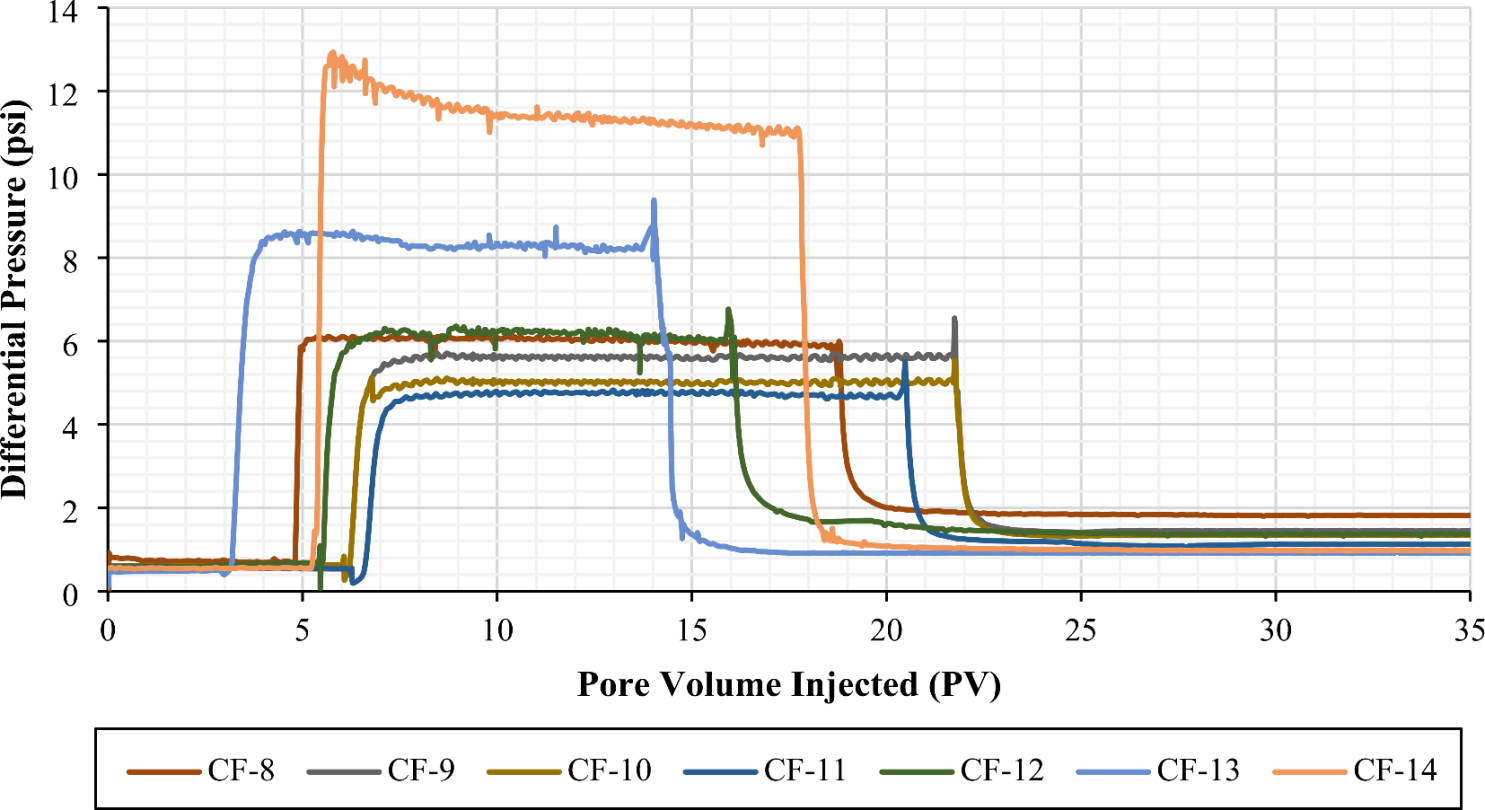
Differential pressure profile for brine pre-flush, polymer injection, and brine post-flush for CF-8 to CF-14 (Two-phase coreflooding experiments). The figure was created using Microsoft Excel (Product name: Microsoft 365, Version number: Version 2409 (Build 18025.20104, 64-bit)).

*Effect of Make-up Water Dilution on Residual Resistance Factor and Adsorbed Layer Thickness in the Absence and Presence of Oil*. From Figs. 7 and 8, it is evident that the differential pressure of post-brine injection at a flow rate of 0.5 cc/min is higher than that of pre-brine injection. This indicates a reduction in permeability, mainly due to the polymer adsorption onto the pore walls. The decrease in permeability can be inferred from the residual resistance factor (RRF). It is worth mentioning that the differential pressure during polymer injection remained stable after the first few pore volumes in all coreflooding experiments, suggesting no significant mechanical entrapment or injectivity issues. Mechanical entrapment would occur if the hydrodynamic radii of the polymer molecules were comparable to or greater than the average pore radius of the porous media. However, the stable pressure readings during polymer injection indicated that the hydrodynamic radii of the polymer molecules were smaller than the average pore radius of the porous media^57^.

**Fig. 9:**
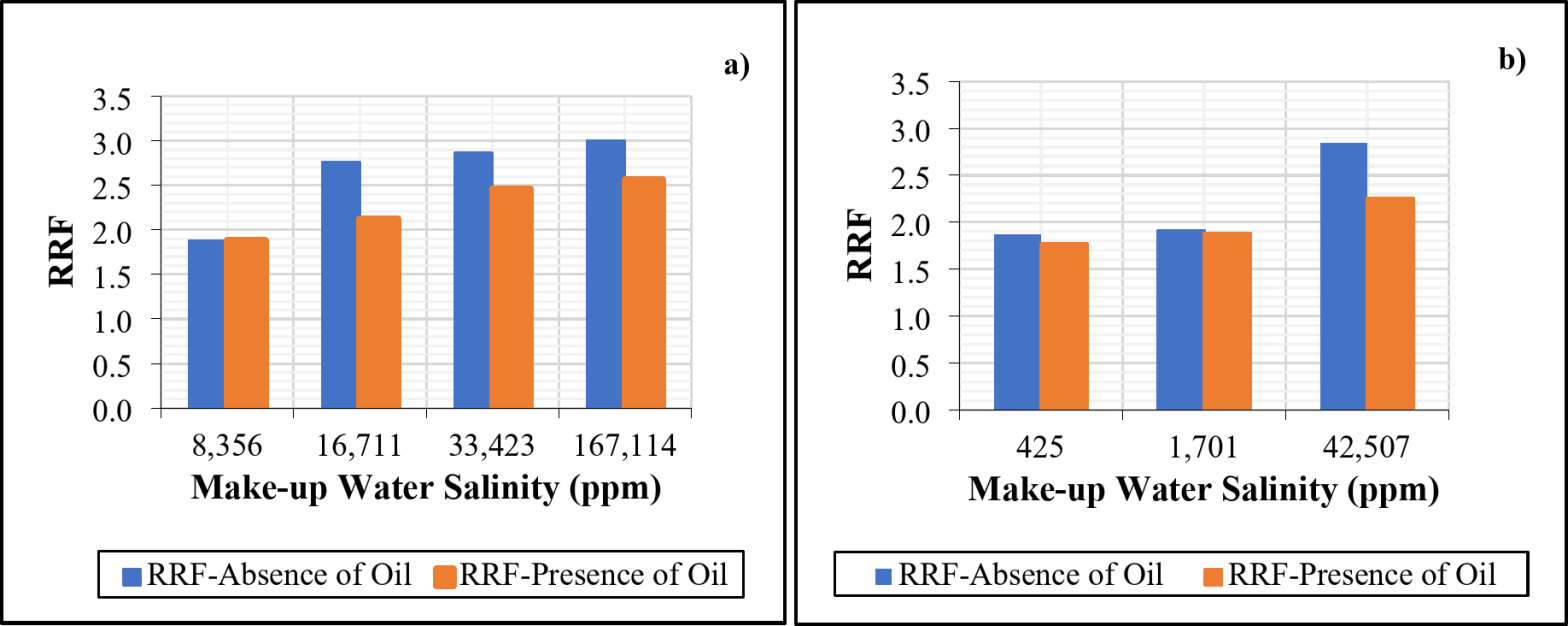
Summary of Residual Resistance Factor (RRF) values caused by polymer injection (a) Formation water and its dilutions, (b) Seawater and its dilutions. The figure was created using Microsoft Excel (Product name: Microsoft 365, Version number: Version 2409 (Build 18025.20104, 64-bit)).

**Fig. 10:**
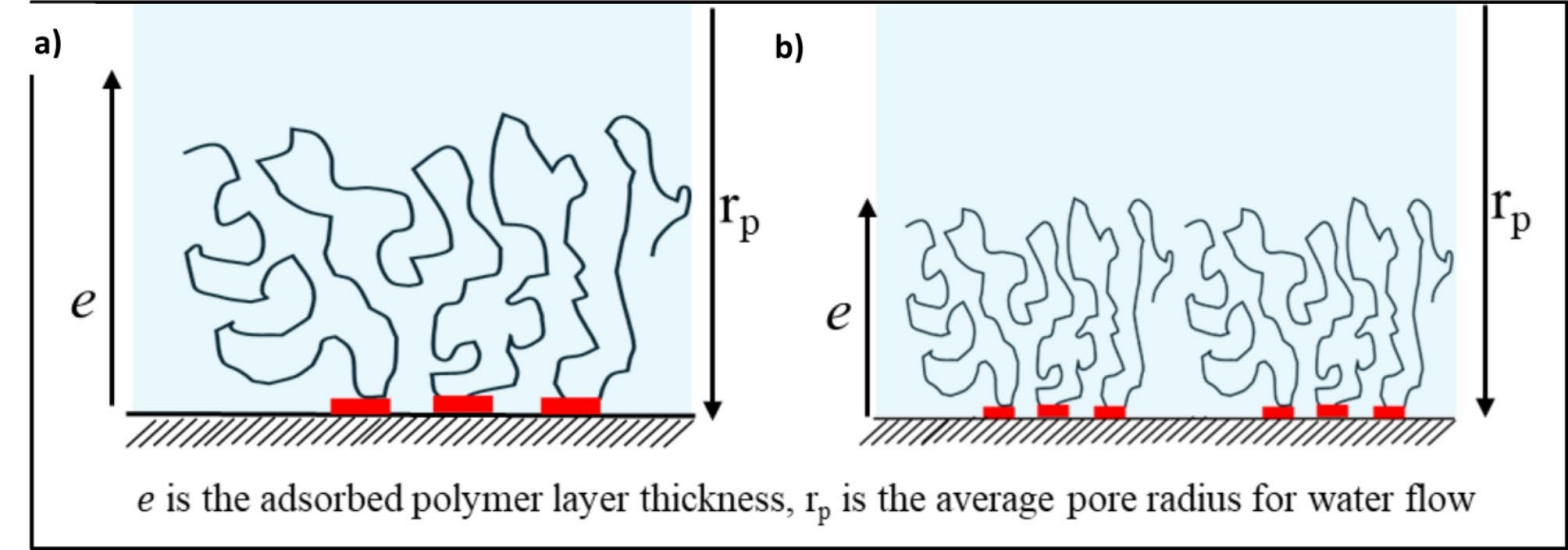
Illustrative representation of the adsorbed polymer layer thickness for a) polymer molecules with larger hydrodynamic size in low salinity brine and b) polymer molecules with smaller hydrodynamic size in high salinity brine (adapted from^27^). The figure was created using Microsoft PowerPoint (Product name: Microsoft 365, Version number: Version 2409 (Build 18025.20104, 64-bit)).

Figure 10 summarizes the RRF values calculated at an injection rate of 0.5 cc/min (approximately 10 ft/d). According to Fig. 10a, the RRF values obtained from polymer flooding experiments with salinities of 167,114, 33,423, 16,711, and 8,356 ppm (formation water and its dilutions) in the absence of oil were 3.00, 2.87, 2.76, and 1.88, respectively. In the presence of oil, the RRF values were 2.56, 2.46, 2.12, and 1.88, respectively. A clear trend emerges, showing lower permeability reduction for low-salinity polymer flooding compared to high-salinity polymer flooding in both the presence and absence of oil. This trend aligns with the lower polymer retention observed in low-salinity polymer flooding compared to high-salinity polymer flooding (Fig. 7a).

**Fig. 11:**
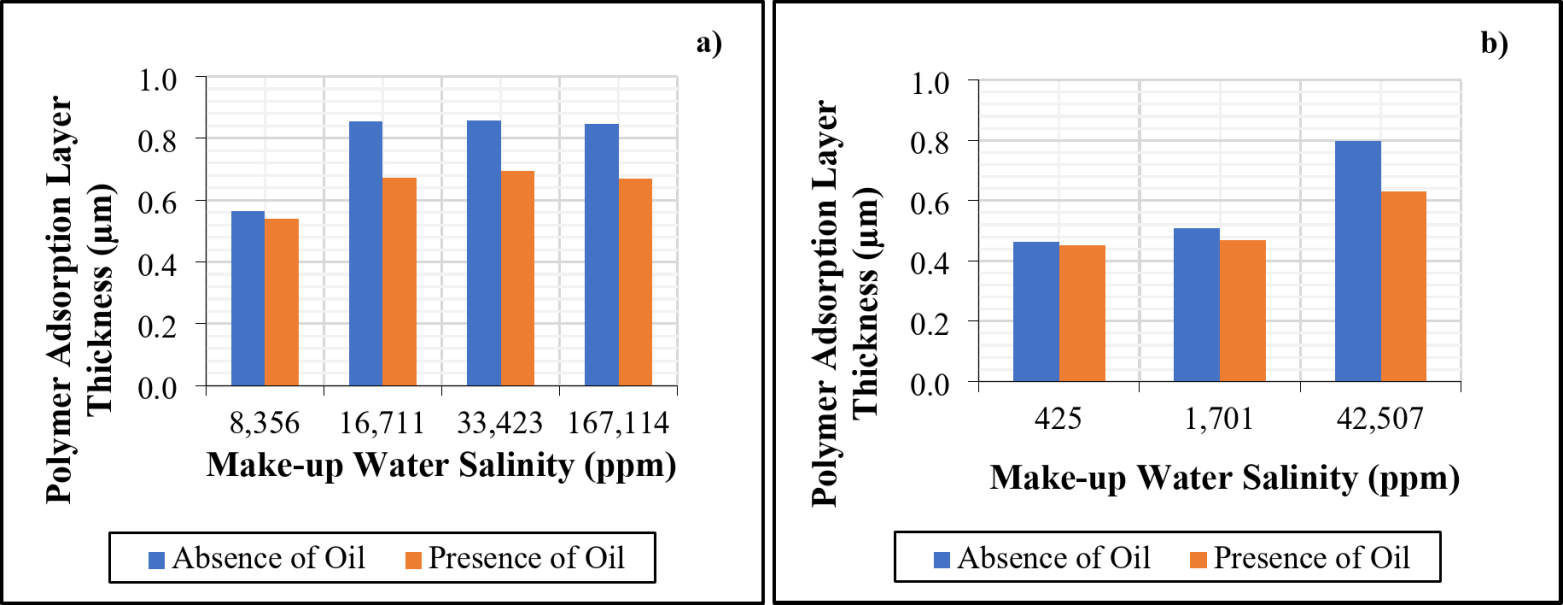
Summary of polymer adsorption thickness due to polymer retention (a) Formation water and its dilutions, (b) Seawater and its dilutions. The figure was created using Microsoft Excel (Product name: Microsoft 365, Version number: Version 2409 (Build 18025.20104, 64-bit)).

When comparing the RRF values in the absence and presence of oil for higher salinities (167,114, 33,423, and 16,711 ppm), we observe a decrease in RRF in the presence of oil, which is consistent with lower polymer retention under these conditions. However, in the low-salinity case (8,356 ppm), the RRF values remain comparable between the absence and presence of oil, while the average polymer retention is lower in the presence of oil than in its absence (Fig. 7a). This can be explained using Eq. (4) from the manuscript, where the average pore radius for water flow is proportional to the brine permeability. For the calculation of RRF values in coreflooding experiments with oil present, the effective brine permeability, which is lower than the absolute brine permeability, is considered. This indicates that the flow cross-section available for the water phase becomes smaller in the presence of oil^65–68^. As a result, even though fewer polymer molecules are adsorbed, the reduction in permeability is more pronounced. This effect is particularly significant in low-salinity polymer solutions because the polymer molecules adopt a larger conformation (increased hydrodynamic size) at lower salinities, as shown in Fig. 11, which increases the thickness of the adsorbed layer^27,69^, thus showing a comparable RRF in the presence and absence of oil (Fig. 10a).

A similar trend was observed for seawater and its dilutions. For low-salinity polymer flooding with salinities of 1,701 and 425 ppm, the RRF values were comparable in the absence and presence of oil. However, in high-salinity polymer flooding (42,507 ppm), the RRF showed a distinct difference between experiments conducted in the absence and presence of oil (Fig. 10b).

The adsorption layer thickness was calculated using Eq. (3) and is depicted in Fig. 11, which corresponds with the RRF trends observed in Fig. 10. This consistency highlights the correlation between adsorption layer thickness and the RRF values, further supporting the overall findings.

## Summary and conclusions

This study investigated the effects of low salinity on polymer viscosity and retention in the presence and absence of oil by conducting 14 coreflooding experiments. The study included both single-phase and two-phase experiments, leading to several significant findings as follows:

- The diluted brines increased the viscosity favorably compared to high salinity injection waters. This could lead to a reduction in polymer flood operating costs by allowing for a lower polymer dosage.
- The study also found that polymer dynamic retention levels were reduced to almost half when diluted brines of salinity less than 10,000 ppm were used, as opposed to high salinity formation water or seawater. While working on polymer injection in the presence of oil, it is noted that the presence of oil in aged cores further reduced polymer retention, emphasizing the importance of using wettability-restored cores to obtain accurate retention values.
- The study also observed that the adsorption mechanism was prominent for all types of brines used since the residual resistance factor (RRF) was less than 3, and there were no undesired pressure peaks during and after polymer injection. This critical finding reveals that the retention-related pressure hikes were absent during low-salinity injection despite having higher polymer viscosity.
- This study shows that low-salinity brine is helpful for polymer flooding and can effectively be used in carbonate reservoirs.

## Future studies

Oil recovery studies will be conducted using low salinity polymer flooding considering the seawater, the formation water, and their various dilutions. Also, further experiments will be conducted to highlight the effects of low salinity water and polymer viscosity on polymer retention; in particular, investigating the low salinity water cut-off for the lowest polymer retention, and decoupling viscosity effect from adsorption by utilizing similar polymer viscosity with varying polymer concentrations in low salinity water dilutions for different adsorption studies.

## Data Availability

All data generated during this study are included in this article. The datasets used and/or analyzed during the current study are available from the corresponding author upon reasonable request.

## Abbreviations

ATBS: Acrylamido-Tertiary-Butyl Sulfonate
CF: Coreflood
EOR: Enhanced Oil Recovery
FW: Formation Water
HPAM: Hydrolyzed Polyacrylamide
NVP: N-Vinyl Pyrrolidone
PV: Pore Volume
RRF: Residual Resistance Factor
SW: Seawater
UV: Ultraviolet
TOC-TN: Total Organic Carbon-Total Nitrogen

## Symbols

C: Concentration (ppm)
K: Permeability (mD)
S: Saturation
V: Volume (ml)
W: Weight (gram)
∆P: Pressure drop (psi)

## Subscripts / superscripts

e: Effluent
i: Initial
or: Residual oil
wi: Initial water
